# An acceptability study of the introduction of total online or partial online PBL in a large classroom setting in biochemistry

**DOI:** 10.1186/s12909-023-04767-3

**Published:** 2023-11-30

**Authors:** Suyun Bai, Hanming Jiang, Tao Wang, Duxiao Yang, Yizhi Liu, Changqin Xu, Limin Zhang, Yuanying Zhang

**Affiliations:** 1 Department of Biochemistry and Molecular Biology, School of Clinical and Basic Medical Sciences, Shandong First Medical University & Shandong Academy of Medical Sciences, Jinan, China; 2 Department of Medical Statistics, School of Public Health, Shandong First Medical University & Shandong Academy of Medical Sciences, Jinan, China; 3 Department of Gastroenterology, Shandong Provincial Hospital Affiliated to Shandong First Medical University, Jinan, China; 4 Department of Rheumatology and Immunology, Shandong Provincial Hospital, Shandong First Medical University, Jinan, China

**Keywords:** Total online PBL, Partial online PBL, Large classroom setting, Undergraduate medical education, Biochemistry

## Abstract

**Background:**

Traditional problem-based learning (PBL) relying on tutored learning in small groups is very resource-intensive. Little is known about the benefits of PBL in a large classroom setting. This paper introduced a PBL case into the traditional didactic biochemistry course and investigated the acceptability of total online or partial online PBL in a large classroom setting introduced during the coronavirus pandemic.

**Methods:**

The students were allocated into either total online Group 1, partial online Group 2, or partial online and with poorer academic performance Group 3. A questionnaire comprising of 8 closed-ended questions and 2 open-ended questions and final exam performances were used to evaluate the acceptability of total online or partial online PBL in a large classroom setting. The 8 closed-ended questions were analysed by the Kruskal–Wallis test or chi-square tests. The word cloud analysis of the 2 open-ended questions were conducted by Wenjuanxing. Students’ performances in the final examination were analysed by One-way Anova.

**Results:**

Both total online and partial online PBL were rated highly by the students. Overall, there were no significant differences in the effectiveness evaluation of PBL between Group 2 and Group 3. There were no significant differences in final exam performances between Group 1 and Group 2. However, Group 1 rated the effectiveness of PBL much higher than Group 2 and 3. Word cloud analysis of the 2 open-ended questions showed students’ positive perspectives of PBL. In biochemistry teaching, from the perspective of the students, the expected optimal number of useful PBL cases might be 2.

**Conclusions:**

Both total online and partial online PBL in a large classroom setting were widely accepted as a beneficial supplement to traditional biochemistry classes.

**Supplementary Information:**

The online version contains supplementary material available at 10.1186/s12909-023-04767-3.

## Background

Problem-based learning (PBL) originated in the Medical School of McMaster University in Canada in 1969 is a student-centered learning approach, in which problems drive the learning [1]. It usually involves learning in small groups to study practical clinical cases and solve medical problems, which are supervised by facilitators [2]. It is accepted that PBL in a small-group setting plays a robust positive role in motivating student learning and cultivating skills required for future professional competence [3, 4]. The methodology and application of PBL are not uniform. Some medical institutions implement Hybrid-PBL curriculum, namely, they implement single or multiple PBL cases within the framework of traditional curriculum [e.g., University of British Columbia Okanagan and San Diego State University], whereas others have carried out whole curriculum reform (e.g., Medical School of McMaster University, Case Western Reserve University and Harvard University) [5–8]. Despite the diversity, it is generally accepted that the primary defining feature of PBL is the contextualization of studying problems presented to students who may have limited preparatory studies in the relevant subject [5].

Biochemistry is a mandatory course for medical undergraduates. However, students usually have significant difficulties in understanding and comprehending of the complicated basic medical science covered by it [9]. Moreover, it is also one of the basic medical sciences that the undergraduates find limited clinical relevance [10]. Therefore, medical biochemistry teachers must highlight how biochemical principles are applied to clinical practice, for example, etiology, pathogenesis, diagnosis, treatment, prognosis, prevention, and health education on diseases thus arousing students’ interest and enhancing the long-term retention of the knowledge. Furthermore, as the general ultimate optimal goal of education, the goal of biochemistry teaching should also be to help the students acquire lifelong learning ability to provide the best and up-to-date care for their future patients [11].

In biochemistry, knowledge of metabolic networks is essential and most challenging. To explain various phenomena under normal and pathological conditions, medical undergraduates must understand the composition and interactive relationships of metabolic networks [12]. It is recognised by numerous professional societies that most undergraduate biochemistry students only gain a superficial level of understanding of metabolism [13]. In the course, the metabolism of carbohydrates, lipids, amino acids, and nucleic acids is mandatory. At our university, the biochemistry of the liver is also covered in the course. The biochemistry of the liver involves the metabolic function, biotransformation of the liver, bile acid metabolism, and bilirubin metabolism. To improve the experience of the teaching-learning process of metabolism biochemistry, we developed a Hybrid-PBL program that combined student-centered PBL with conventional lectures, which would equip future doctors with the knowledge and skills required for their future careers.

Currently, there is a paucity of research about the benefits of total online and partial online Hybrid-PBL in a large classroom setting. In this research, we showed and discussed the results obtained from the application of total online and partial online large class PBL case scenario sessions integrating amino acid metabolism and the biochemistry of the liver during the coronavirus pandemic.

## Materials and methods

### Participants and study setting

In the first semester of their second academic year, 689 (100%) medical undergraduates at our university were enrolled during their regular biochemistry classes in this study. All the students had no prior experience with PBL in their medical courses. The global coronavirus disease (COVID-19) pandemic forced universities in China to conduct partial or total online teaching. In this study, the students were grouped according to their learning performances and the pandemic control regulations: Group 1 is a total online group, Groups 2 and 3 are two partial online groups (namely the first session is a face-to-face one, but the second is online), with 327, 163 and 199 students in the three groups, respectively. There were no gender or age differences between the three groups. For the first two groups, there were also no academic performances differences before the start of the course. The students of the third group are from a special group in China. They are known as public-funded students, and most of them will be assigned to township hospitals after graduating from university. It was known that the learning ability and motivation of the first two groups were significantly higher than those of the third group. The three groups were further randomly divided into many subgroups. Each PBL subgroup constituted ten to eleven students with a chair and a recorder to organise case discussions and record the discussion processes. The facilitators responsible for instructing PBL groups were biochemistry teachers. The PBL case scenario sessions were conducted in large classrooms with approximately 5 subgroups in each classroom. The online and face-to-face PBL case scenario sessions were guided by a facilitator via video conference and face-to-face, respectively. For both online and face-to-face PBL sessions, the ratio of facilitators to subgroups was one facilitator per the 5 subgroups.

### PBL arrangements

After traditional lectures about carbohydrate and lipid metabolism were finished, the students were offered some online resources including learning objectives, PPT, and micro-lectures to study amino acid metabolism and the biochemistry of the liver by themselves. Then a PBL case about alcoholic liver, which involved the progression of alcoholic fatty liver to alcoholic hepatitis and then, at last, hepatic encephalopathy, was offered to the students. The case was modified from real cases by a trained PBL writing team with biochemistry teachers and clinical experts to suit the learning objectives of the curriculum. While constructing the case, the team members constructed a detailed tutor’s guide. PBL case scenario experts of our college approved the case. The case covered most key knowledge about amino acid metabolism and the biochemistry of the liver. In addition, it also involved some knowledge about carbohydrates metabolism, lipids metabolism, and the cross-talk of the metabolism of the three nutrients. The case was designed for two sessions with each lasting 2.5 h. Training sessions for the teaching faculty and questionnaire were standardised before the research. Due to the students’ having no prior experience with PBL, before the first PBL session, they were trained on PBL philosophy and general procedures. Senior students with rich PBL experience shared their experience. To help the students determine the reliability and feasibility of the resources acquired from the network in the following sessions, evidence-based medicine (EBM) philosophy which is the use of high-quality clinical research to determine the care of patients was also introduced. In addition, in order to promote the learning in PBL sessions, a learning contract which stated the responsibilities of the tutor, chair, recorder and other student members was signed. During each PBL session, the students were required to identify essential clues or problems, list hypotheses, list proofs supporting the hypotheses, generate a series of learning issues and learn the issues from biochemistry and other medical disciplines’ perspectives. The students were advised to access online resources and refer to textbooks, including biochemistry, diagnostics, internal medicine, etc. The recorders recorded the sessions, and the contributions of all the members in every subgroup were marked. After each session, all subgroups were required to submit a report containing the four items mentioned above to the facilitator. Before and after the second session, the facilitators commented on the reports of the first and second sessions respectively, praised the strengths of each group, pointed out their shortcomings, and gave some suggestions for improvement. At last, the facilitators concluded the key learning objectives and the relevant biochemical knowledge hidden in the case.

### Survey of students

Based on previous studies [6, 14–16], an online survey platform called Wenjuanxing (www.wjx.cn) was used to develop a questionnaire comprising of 8 closed-ended questions and 2 open-ended questions for the analysis of students’ perceptions of the effectiveness of the PBL case scenario sessions. The survey had two spaces for their free response comments on the following 2 open-ended questions: (a) What have you learned the most from PBL? (b) What advantages and disadvantages do the PBL sessions have compared with traditional teaching methods? What do you think the current PBL sessions need to be improved?

The evaluations were completed immediately after the case scenario sessions. Respondents scored the 6 survey items in Table A 2(Additional file 1) on a 4-point rating scale, with 0 = *strongly disagree or disagree*, 1 = *neutral*, 2 = *agree*, and 3 = *strongly agree*. The participation of students in the survey was voluntary and the anonymous nature of their responses was assured.

### Appraisal of the learning effectiveness of PBL in the final examination

The learning effectiveness of PBL was appraised in the in-person final written biochemistry examination. The examination questions included Multiple Choice (40 marks), True or False (10 marks), Terminology (24 marks), and Essay Questions (26 marks). The last essay question was a 6-point question that asked the students to write the first scenario of a PBL case, extract relevant biochemistry problems from it and then explain the problems. Besides the PBL case essay question, there were also some other questions related to the PBL case they had studied, including 1 Multiple Choice and 4 True or False questions. These questions were used to evaluate the learning effectiveness of the PBL case scenario sessions.

### Statistical analysis

SPSS for Windows, version 25 (IBM SPSS), was applied for all analysis. The 8 items were analysed by the Kruskal–Wallis test or chi-square tests. In addition, comparisons of the performances of the students including PBL case related question marks and total scores in the final examination between the three groups were analysed by One-way Anova.

## Results

### Student performances in the PBL sessions

In the PBL sessions, with the guidance of the facilitators, students from the 3 groups worked actively and excitedly together to analyse the case scenarios, identify important clues, make their preliminary disease judgments, list proofs to support their judgements, discuss the hidden learning issues related to medical sciences mainly biochemistry, ‘population’ health and humanistic care.

### Student perceptions of PBL Sessions

Among the 689 clinical medical undergraduates, 685 (99.42%) students completed the questionnaire. Table A1(Additional file 1) showed students’ evaluations of the overall effectiveness of PBL case scenario sessions. All the three groups of students rated the PBL sessions highly. Students of Group 1 rated the PBL sessions higher than those of Group 2 and Group 3 (*p* < 0.001). There was no evaluation difference between Group 2 and Group 3 (*p* > 0.05).

Table 1 showed that no difference in expectation for the number of PBL cases that the students perceived to be useful for learning in the biochemistry curriculum was noted between the three groups (*p* > 0.05). Based on the data in Table 1, the ideal number might be 2.

**Table 1:** Students’ expectations for the number of perceived useful PBL cases in biochemistry curriculum

Group	Students’ expectation number					*χ2*	*P*- value
	0	1	2	3	>3		
Group 1, *n* (%)	15(4.63)	103(31.79)	114(35.19)	44(13.58)	48(14.81)	8.422	>0.05
Group 2, *n* (%)	11(6.75)	57(34.97)	54(33.13)	24(14.72)	17(10.43)		
Group 3, *n* (%)	16(8.08)	51(25.76)	66(33.33)	33(16.67)	32(16.16)		

Notes: Analysed by means of the Pearson chi-square tests

Table A2 (Additional file 1) depicted students’ perceptions of PBL in cultivating their self-directed learning skills. Most students from the 3 groups believed that they had obtained more abilities from PBL than from conventional learning methods. Data from Table A2 showed the ratings of students on the 6 items, with the mean ± SD ranging from 2.06 ± 0.82 to 2.71 ± 0.48 (based on the 4-point rating scale mentioned above). Moreover, students of Group 1 showed higher ratings on all the 6 items than Group 2 and Group 3 (*p* < 0.001, *p* < 0.01, or *p* < 0.05). Except for the item ‘beneficial to develop deeper self-awareness’, no difference between Group 2 and Group 3 was noted.

‘Wenjuanxing’ is a professional online questionnaire survey platform widely used in China. It can automatically extract the keywords contained in all the answers of the two free-response comment questions and automatically classify and analyse them to provide word clouds. A word cloud, a form of data visualization, is a graphic representation of words or phrases that gives greater prominence to those that appear more frequently [17]. The hot words or phrases are the high frequency words or phrases in the word clouds. Our content analysis validated the results provided by this platform. For the first question about the most significant benefit from PBL learning, the hot words or phrases are ‘self-directed learning’, ‘collaborative learning’, ‘integration’, ‘deepen understanding’, ‘presentation skills’, ‘collecting information’, ‘brainstorming’, ‘clinic ideation’, ‘contextualised learning’. Combination of the hot words with content analysis of the answers of the second question was helpful to distinguish the advantages and disadvantages of PBL. Results showed that the advantages of PBL partially overlapped with those in the first question, for example, ‘self-directed learning’, ‘collaborative learning’, ‘clinic ideation’. The hot words of the second question also showed us some shortcomings of the current PBL, for example, ‘less efficient than traditional learning methods’, ‘insufficient knowledge reserve’, ‘excessive number of students per subgroup’, ‘difficulty grasping the key knowledge in time’.

### Appraisal of learning effectiveness of PBL by academic performances

Table A3 showed that there were no academic performance differences between Group 1 and Group 2. In addition, as we expected, the academic performance of the Groups 1 and 2 was significantly higher than Group 3.

## Discussion

The essence of PBL is its connotation and theoretical principles of learning which include contextualised learning, constructive learning, collaborative learning, and self-directed learning. These elements lead to its unique advantages over traditional didactic teaching methods. However, traditional PBL relying on tutored learning in small groups is very resource-intensive. This has seriously hindered the implementation of PBL in many medical schools, including most medical schools in China. Therefore, the study of large class PBL is of great significance. To our knowledge, this is the first study to explore the feasibility of the introduction of total online or partial online large class PBL in biochemistry course. Our questionnaire and final exam analysis indicated that both total online and partial online PBL in a large classroom setting were considered as a beneficial complement to traditional biochemistry teaching. The cultivation of students’ abilities and skills is a long-term process. Therefore, we cannot expect one or two PBL cases to significantly improve students’ multifaceted abilities and skills. However, PBL undoubtedly gives students a learning idea and approach different from traditional teaching.

A few studies had validated the feasibility of different approaches to face-to-face large class PBL. Qiu Yan et al [2] had successfully introduced the Hybrid-PBL curriculum in biochemistry in a large classroom facility. However, there were some differences between their study and ours. The main differences were as follows: (a) their students were international medical students with diverse educational backgrounds; (b) their PBL cases were introduced after the relevant lecture were completed, thus emphasizing the application of knowledge and paying less attention to the construction of knowledge; (c) their PBL cases were simple cases which were something like CBL (case-based learning) cases; (d) their PBL case scenario sessions were face to face. Sheffield Medical School in the UK evaluated the introduction of ILA (integrated learning activity) format face-to-face large class PBL, a not full PBL approach, into an undergraduate medical curriculum of first-year students, which showed that there seemed to be no significant difference in learning outcomes between small group teaching format and ILA format [18]. A study from China Medical University also showed that the application of another form of face-to-face large class PBL in stomatology course is feasible [19].

However, some factors restricted the implementation of large class face-to-face PBL, for example, pandemics, cross-regional collaborative PBL or others. Our questionnaire survey and final examination showed that most enrolled students had deeply understood and experienced the essence of PBL. Therefore, when face-to-face large class PBL is restricted, total online and partial online PBL might provide a new alternative approach to PBL implementation.

Both the survey about students’ expectations for the number of PBL cases and word-cloud analysis of the second open question indicated that most students were accustomed to traditional didactic teaching, which was considered as the most efficient way to acquire knowledge in a short term. Therefore, too many PBL sessions may burden the students and not be conducive to the current academic situation. Jie Li et al. [20] claimed that the following factors might explain the failure of PBL implementation in Asian countries: (1) students were accustomed to traditional teaching methods, which might have changed their learning style dramatically, affecting their perceptions and learning effectiveness; (2) student learning effectiveness in PBL might be affected by features of the specific subject itself; (3) sampling bias resulting from tiny sample sizes. Therefore, the claims of Jie Li also explained our large sample study results.

Tutor quality, student motivation, and realistic cases are the three known difficulties that strongly influence the effect of PBL, and these factors vary over a broad range [21]. In our study, based on real clinical cases, a case that implied much biochemical knowledge was designed by a team of biochemical teachers and clinical experts with rich PBL case scenarios writing experience. Most students in our university are from Shandong province, which has a long and robust liquor culture. Therefore, our cases gave students a realistic feeling close to life. In this PBL, the main learning objective of ‘life sciences’ was set as discovering the relevant biochemical mechanisms and the integration of biochemistry with other disciplines. In addition, a detailed tutor guidebook was handed to all the tutors whom experts with rich tutor experience had trained. Although tutor quality and a realistic case were standardised and unified in this study, the three groups of students might vary in their motivation. This might have some impact on students’ perceptions. In our study, the evaluation of students of Group 1 who had taken longer online courses was higher than Group 2 and Group 3.

The worldwide lockdown restrictions resulting from the COVID-19 pandemic impacted all aspects of life, including the sudden shift from face-to-face teaching to online teaching. Research on the perceptions of 2721 medical students across 39 medical schools from the UK during the pandemic indicated the advantages and disadvantages of online teaching and students’ preference for face-to-face teaching [22]. Advantages of online teaching include saving students’ time on travelling, more comfort, cutting costs, and providing the flexibility and opportunity for students to learn at their own pace [22]. The disadvantages are as follows: unstable internet connection, anxiety, lack of contact with colleagues, lack of motivation, difficulty concentrating and asking questions, etc. [22]. Due to disrupted social connections and confinement measures, virtual learning also caused increased levels of loneliness and social isolation [23]. In addition, the exaggerated videoconferencing use results in a widespread negative effect of fatigue or videoconference exhaustion, with a general average prevalence of 48% [24]. The Attention Restoration Theory (ART) of Kaplan explains how human energy is consumed. It postulates that if individuals are in meetings where they have the perception of belonging to the group (‘compatibility’), they will feel more connected with each other and motivated (‘soft fascination’), and this reduces the degree of fatigue [25]. PBL, as an active methodology, reduced online learning fatigue among medical students [24]. In our study, there were no significant academic performances differences in the final examination between Group 1 and Group 2. Therefore, the factors mentioned above might explain the higher evaluation of Group 1.

The hot words analysis about the shortcomings of the current PBL pointed the way for further improvement. Optimizing the internet resources available to the PBL program, group staffing, and timely guidance by tutors should be carried out in the future.

### Limitations

This research has some limitations. It is only a preliminary study on the role of total online or partial online large class PBL introduced in biochemistry, with only one PBL case scenario involved. In the future, more detailed and comprehensive studies should be carried out, including other PBL case scenarios that involve other diseases and some related knowledge, the more precise appropriate timing of PBL introduction in biochemistry, the optimizing of group staffing and more timely and effective guidance to maximize the benefits for as many students as possible, etc. Furthermore, all the students enrolled in this study had no prior PBL experience in all their medical courses. Moreover, the curriculum also included lecture content on the theory assessed in the exam. All these factors may modify the effect of PBL.

## Conclusions

In this study, we successfully combined total online or partial online PBL in a large classroom setting with conventional biochemistry lectures. Our medical undergraduates well accepted total online or partial online PBL as an effective supplement to traditional classes. Our study was an innovative trial that introduced total or partial online PBL in a large classroom setting to medical undergraduate students in a specific discipline. This might provide a perspective for the universal use of PBL in many other disciplines. Moving forward from the pandemic, to improve the efficacy of medical biochemistry teaching, especially the efficacy of online teaching during future possible pandemics or cross-regional collaborative teaching, we suggest that medical school resort to online or partial online PBL introduction in the biochemistry curriculum.

### Electronic supplementary material

Below is the link to the electronic supplementary material.

Supplementary Material 1

## Data Availability

The datasets used and/or analysed during the current study are available from the corresponding author upon reasonable request.

## List of abbreviations

PBL problem: based learning
EBM evidence: based medicine
CBL case: based learning
ILA: integrated learning activity
ART: Attention Restoration Theory
