# Supplementary material for: An acceptability study of the introduction of total online or partial online PBL in a large classroom setting in biochemistry
Source: BMC Med Educ. 2023 Nov 30;23:912. doi: 10.1186/s12909-023-04767-3 (PMC10691088; doi:10.1186/s12909-023-04767-3)
Supplement: Supplementary file 1 — Supplementary Material 1 [file 12909_2023_4767_MOESM1_ESM.docx]

**Table A1.** Students’ overall evaluations of the effectiveness of the PBL case scenario sessions

| **Group** | **Students’ evaluations** | | | | | ***P*- value** | | | |
| --- | --- | --- | --- | --- | --- | --- | --- | --- | --- |
|  | **Poor** | **Fair** | **Average** | **Good** | **Excellent** | **G1 vs G2 vs G3** | **G1 vs G2** | **G1 vs G3** | **G2 vs G3** |
| G1, n (%) | 6 (1.85) | 1 (0.31) | 44 (13.58) | 103 (31.79) | 170 (52.47) | < 0.001 | < 0.001 | < 0.001 | > 0.05 |
| G2, n (%) | 2 (1.22) | 6 (3.68) | 39 (23.93) | 79 (48.47) | 37 (22.70) |  |  |  |  |
| G3, n (%) | 3 (1.52) | 10 (5.05) | 38 (19.19) | 81 (40.91) | 66 (33.33) |  |  |  |  |

Notes: Analysed by means of the Kruskal–Wallis test

G1, G2 and G3 represent groups 1, 2 and 3 respectively

**Table A2.** Comparison of students’ ratings of the effectiveness of PBL in cultivating their self-directed learning skills between the three groups (mean±SD)

| **Items** | **G1** | **G 2** | **G 3** | ***H*-value** | ***P*-value** | | | |
| --- | --- | --- | --- | --- | --- | --- | --- | --- |
|  |  |  |  |  | **G1 vs G2 vs G3** | **G1 vs G2** | **G1 vs G3** | **G2 vs G3** |
| 1. Being helpful to promote the integration of knowledge (both within biochemistry discipline and with other disciplines) and learn about the frontiers of knowledge | 2.65±0.52 | 2.40±0.59 | 2.52±0.57 | 23.50 | < 0.001 | < 0.001 | < 0.01 | ＞0.05 |
| 2. Greatly improved the ability to collect, re-organise, and analyse information and presentation skills | 2.71±0.48 | 2.51±0.57 | 2.52±0.57 | 20.89 | < 0.001 | < 0.001 | < 0.01 | ＞0.05 |
| 3. Beneficial to cultivate interpersonal skills and teamwork ability | 2.48±0.71 | 2.22±0.73 | 2.28±0.82 | 19.97 | < 0.001 | < 0.001 | < 0.05 | ＞0.05 |
| 4. Beneficial to develop deeper self-awareness | 2.45±0.74 | 2.06±0.82 | 2.27±0.76 | 31.30 | < 0.001 | < 0.001 | < 0.01 | < 0.05 |
| 5. Beneficial to think medical humanities, health care, and disease prevention thoroughly, thus developed professionalism | 2.53±0.68 | 2.23±0.72 | 2.34±0.71 | 27.15 | < 0.001 | < 0.001 | < 0.01 | ＞0.05 |
| 6. Beneficial to develop leadership | 2.47±0.73 | 2.09±0.87 | 2.27±0.80 | 27.33 | < 0.001 | < 0.001 | < 0.01 | ＞0.05 |

Notes: Analysed with the Kruskal–Wallis test.

G1, G2 and G3 represent groups 1, 2 and 3 respectively

**Table A3**. Comparison of students’ performance in the final exam between the three groups (mean±SD)

| **Students’ performance 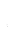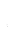** | **G1**  ***n*=327** | **G2**  ***n*=163** | **G3**  ***n*=199** | ***P*- value** | | | |
| --- | --- | --- | --- | --- | --- | --- | --- |
|  |  |  |  | **G1 vs G2 vs G3** | **G1 vs G2** | **G1 vs G3** | **G2 vs G3** |
| Marks of PBL related questions (0-9) | 6.75±1.58 | 6.94±1.57 | 6.29±1.69 | < 0.001 | > 0.05 | < 0.01 | < 0.001 |
| Total scores (0-100) | 77. 76±13.52 | 77.75±11.96 | 68.18±12.06 | < 0.001 | > 0.05 | < 0.001 | < 0.001 |

Notes: Analysed by means of One-way Anova

G1, G2 and G3 represent groups 1, 2 and 3 respectively
